# Comparison of Oxford Nanopore Technologies sequencing protocols using R9.4.1 and R10.4.1 flow cells under 20-hour and 48-hour conditions for genotyping methicillin methicillin-resistant *Staphylococcus aureus* (MRSA)

**DOI:** 10.1007/s11033-026-11956-y

**Published:** 2026-05-14

**Authors:** Mário Sérgio Braga do Couto, Karin Helmersen, Aluisio Valerio Deola, Glauber Wagner, Hege Vangstein Aamot, Fabienne Antunes Ferreira

**Affiliations:** 1 Bacterial Molecular Genetics Laboratory (GeMBac), Department of Microbiology, Immunology, and Parasitology, Biological Sciences Center, Universidade Federal de Santa Catarina, Campus Universitário Reitor João David Ferreira Lima, Trindade, Florianópolis, 88040-960 SC Brazil; 2 Department of Microbiology and Infection Control, Akershus University Hospital, Lorenskog, Norway; 3 Department of Clinical Molecular Biology (Epigen), Akershus University Hospital, University of Oslo, Lorenskog, Norway; 4 Bioinformatics Laboratory, Department of Microbiology, Immunology, and Parasitology, Biological Sciences Center, Campus Universitário Reitor João David Ferreira Lima, Trindade, Florianópolis, 88040-960 SC Brazil

**Keywords:** Genome analysis, Antibiotic resistance, Nanopore sequencing, Flow cell, MRSA, Whole genome sequencing

## Abstract

**Background:**

Rapid and accurate whole-genome sequencing (WGS) is increasingly important for detecting antimicrobial resistance and virulence determinants in clinical microbiology. Although short-read platforms remain widely used, long-read sequencing technologies have gained relevance due to their ability to resolve complex genomic regions.

**Methods and results:**

We analyzed 92 draft genomes generated from 23 clinical methicillin-resistant *Staphylococcus aureus* (MRSA) isolates to compare Oxford Nanopore Technologies sequencing protocols using R9.4.1 (Kit V10) and R10.4.1 (Kit V14) flow cells under 20-hour and 48-hour conditions. The 20-hour R10.4.1 protocol (ONT20h R10) showed the best overall performance, with improved detection of antimicrobial resistance genes, virulence factors, plasmids, and mobile genetic elements. This protocol achieved perfect agreement with phenotypic antimicrobial susceptibility testing (accuracy 100%, κ = 1.0), supporting its suitability for rapid genomic analysis. Highly repetitive mobile genetic elements remained challenging to fully resolve, reflecting the technical difficulty of assembling complex repeats.

**Conclusion:**

Overall, this study supports the use of rapid long-read sequencing as a complementary approach for clinical microbiology and genomic surveillance, particularly when timely genomic information is required.

**Supplementary Information:**

The online version contains supplementary material available at10.1007/s11033-026-11956-y.

## Introduction

Understanding how bacterial pathogens spread, evolve, and develop resistance is key to guiding treatment decisions and improving public health surveillance [1]. Whole Genome Sequencing (WGS) has revolutionized these investigations, offering a high-resolution view of transmission dynamics, resistance genes, and mobile genetic elements (MGE) that often go undetected by conventional methods [2].

Short-read platforms such as Illumina remain the benchmark for routine use due to their high accuracy, but their inability to assemble complex genes or complete operons, to resolve repetitive regions and fully reconstruct plasmids limits their scope in certain applications [2]. Long-read technologies, particularly those developed by Oxford Nanopore Technologies (ONT), offer an attractive alternative by enabling the generation of complete bacterial genomes in a cost-effective and portable format. Although sequencing errors were initially a major concern, recent improvements in ONT chemistry, flow cell design and basecalling algorithms have led to significant gains in accuracy and expanded the potential for their use in microbial genomics and surveillance [2–4].

A major technological advancement was the 2022 transition from the R9.4.1 to the R10.4.1 flow cell, which incorporated structural innovations such as elongated nanopores and a dual reader head architecture. These modifications improved the resolution of homopolymeric regions and, in conjunction with updated sequencing kits and refined basecalling algorithms, led to a substantial reduction in error rates [5, 6].

We recently sequenced 42 clinical isolates of methicillin-resistant *Staphylococcus aureus* (MRSA) to compare different sequencing protocols for the detection of antimicrobial resistance genes, virulence factors, and MGE [7]. In that study, a 20-hour sequencing protocol using the ONT platform (referred to as ONT20h) demonstrated accuracy comparable to or greater than that of longer protocols for identifying these genetic features. Additionally, ONT20h achieved good concordance between phenotypic and genotypic antimicrobial susceptibility testing (AST) results [7]. All isolates in that study were sequenced using the R9.4.1 flow cell and the ONT-only data showed high concordance with Illumina and hybrid assemblies for detecting genetic markers, establishing it as a robust baseline [7]. In the present study, we selected 23 of those 42 MRSA isolates for resequencing with the R10.4.1 flow cell to evaluate improvements in the detection of genetic elements.

## Materials and methods

As previously described [7], all MRSA isolates used in this study were obtained from the strain collection of the Department of Microbiology and Infection Control, Akershus University Hospital, University of Oslo, Norway. We compared four sequencing protocols that differed in flow cell type (R9.4.1 or R10.4.1), sequencing duration (20–48 h), sequencing chemistry generation (kit V10 or kit V14), and basecaller version (Table 1). All draft genomes were generated using the GridION platform (ONT) and barcode demultiplexing as well as quality control were conducted using the EPI2ME (ONT). Reads with a quality score above 9 were extracted after either 20–48 h of sequencing from the same flow cell for downstream analysis. The genomes sequenced with the R9.4.1 flow cell (ONT20h R9 and ONT48h R9) were previously generated and analyzed by Cipriani et al. [7], while those sequenced with the R10.4.1 flow cell (ONT20h R10 and ONT48h R10) were produced in the present study. For the R10.4.1 flow cell, the DNA sequencing library was prepared using the Rapid Barcoding Kit 24 V14 (SQK-RBK114.24), and real-time basecalling was performed with the Guppy basecaller v7.0.9 (ONT). All the genomes were assembled using Flye [8], followed by two rounds of polishing with Medaka (https://github.com/nanoporetech/medaka). Assembly quality was evaluated with QUAST v5.2.0 [9].

**Table 1:** Overview of sequencing and assembly protocols using GridION from Oxford Nanopore Sequencing (ONT)

Protocol	Flow cell	Sequencing time	Sequencing kit and Basecalling	De novo assembly software tool	Polishing software tool
ONT20h R9	R9.4.1	20 h	Rapid Barcoding Kit (SQK-RBK004) (Kit V10) and Guppy basecaller v3.0.6	Flye v.2.7.1	Medaka v.1.0.1
ONT48h R9	R9.4.1	48 h	Rapid Barcoding Kit (SQK-RBK004) (Kit V10) and Guppy basecaller v3.0.6	Flye v.2.9	Medaka v.1.5.0
ONT20h R10	R10.4.1	20 h	Rapid Barcoding Kit 24 V14 (SQK-RBK114.24) (Kit V14) and Guppy basecaller v7.0.9	Flye v.2.9.3	Medaka v.1.12.0
ONT48h R10	R10.4.1	48 h	Rapid Barcoding Kit 24 V14 (SQK-RBK114.24) (Kit V14) and Guppy basecaller v7.0.9	Flye v.2.9.3	Medaka v.1.12.0

Antimicrobial resistance genes, virulence-associated genes, plasmids, and other mobile genetic elements (MGEs) were identified using tools available at the Center for Genomic Epidemiology (http://www.genomicepidemiology.org): ResFinder v4.7.2 [10], VirulenceFinder v2.0.5 [11], PlasmidFinder v2.0.1 [12], and MobileElementFinder v1.0.3 [13], respectively. All tools were run with default parameters. Among these, only the ResFinder database was updated for the analyses using R10.4.1 flow cell data, in which the version from March 22, 2024, was applied. This update introduced newly described genes and variants but did not alter the core set of resistance determinants (e.g., *mecA*, *blaZ*) present in our MRSA isolates. Therefore, the comparative analysis of detection accuracy for these conserved targets is not biased by the database version difference. The other tools were used with the same database versions as in the R9.4.1 analysis. Genes detected were classified into three categories based on sequence identity and length relative to the reference: Category A includes sequences identical in both identity and length. Category B includes sequences identical in length but with sequence identity between 90 and < 100%. Category C includes sequences with identity between 90 and < 100% and length between 60 and < 100% of the reference. The comparison between phenotypic and genotypic antimicrobial susceptibility testing (AST) was performed using the phenotypic AST data generated previously for these isolates [7]. The statistical analysis followed the same methodology described therein [7]. Only 20 out of the 23 sequenced genomes were included in this comparison, as phenotypic AST data were unavailable for three isolates.

## Results and discussion

In the present study, we sequenced 23 MRSA isolates using the R10.4.1 flow cell and generated 46 draft genomes by analyzing both 20-hour and 48-hour outputs from the same sequencing runs (protocols ONT20h R10 and ONT48h R10). These 46 assemblies were then compared to the corresponding genomes previously sequenced using the R9.4.1 flow cell [7]. Draft genome quality was assessed using standard assembly metrics (Supplementary Material 1). A clear difference in assembly contiguity was observed between flow cell chemistries. Protocols using the R9.4.1 flow cell produced highly contiguous draft assemblies, with an average of 1.8 contigs, whereas assemblies generated with the R10.4.1 chemistry were more fragmented, averaging 17.6 contigs.

This difference is primarily explained by experimental design: R10 flow cells were used to sequence a higher number of samples simultaneously (12 vs. 6 in R9), reducing per-sample throughput and the number of reads obtained per genome. Consequently, R10 assemblies were more fragmented, despite maintaining sufficient coverage for downstream analyses.

**Fig. 1:**
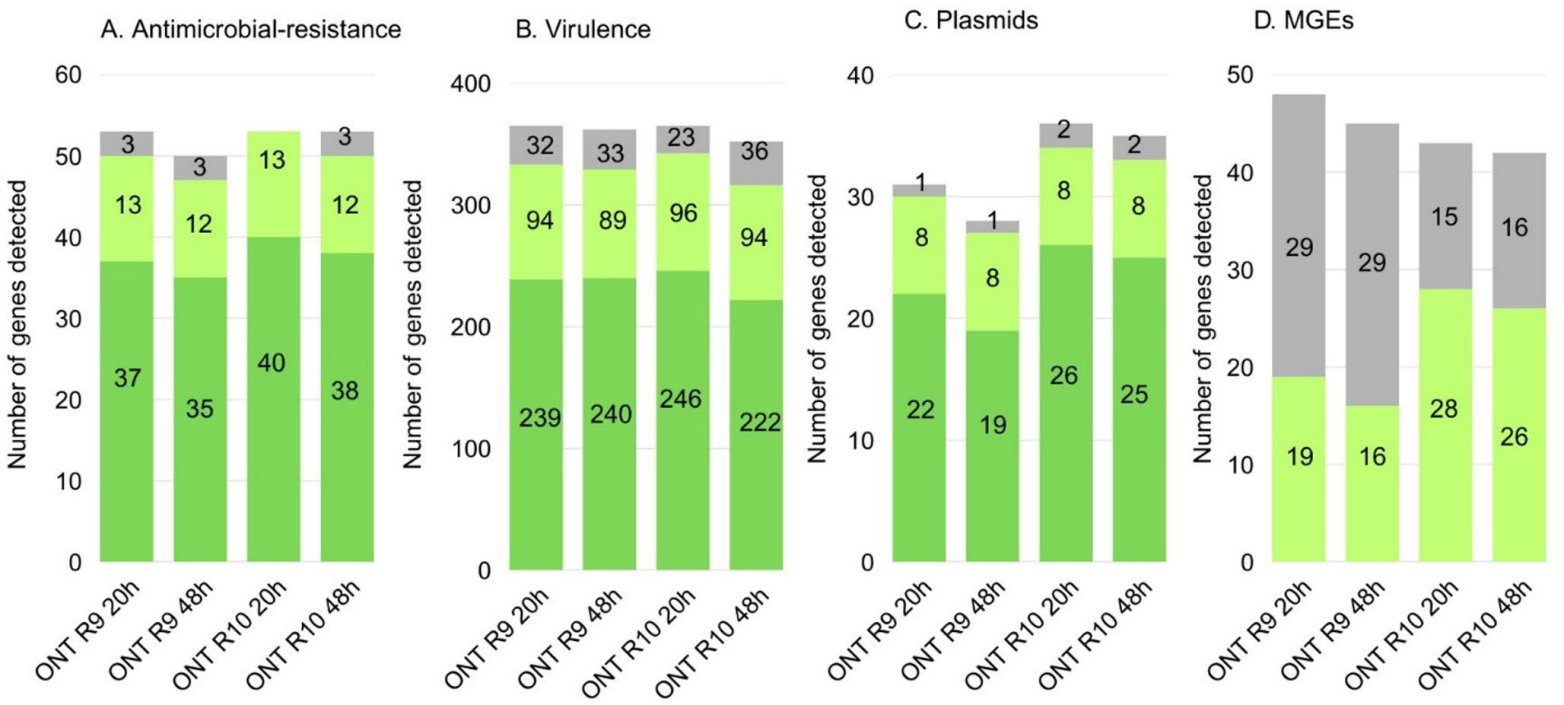
Total number of genes and other genetic elements detected in MRSA (methicillin-resistant *Staphylococcus aureus*) genomes. (**a**) antimicrobial resistance genes; (**b**) virulence-related genes; (**c**) plasmids; (**d**) mobile genetic elements (MGEs). Detected elements were classified into three categories: Category A (dark green), sequence length in the genome identical to the reference with 100% identity; Category B (light green), sequence length identical to the reference with identity between 90 and < 100%; Category C (gray), sequence length between 60 and < 100% of the reference with identity between 90 and < 100%

Importantly, this fragmentation did not negatively impact on the study’s objectives. All genomes achieved adequate coverage for reliable gene detection, and R10.4.1-based protocols, particularly ONT20h R10, demonstrated superior performance in detecting antimicrobial resistance genes, virulence factors, plasmids, and mobile genetic elements (Fig. 1). In the comparative analysis of antimicrobial resistance genes (Fig. 1a), the ONT20h R10 protocol detected the most genes (55), while the ONT48h R9 detected the fewest (50). The ONT20h R10 classified 42 genes (76.4%) in Category A, with no genes in Category C. ONT48h R10 ranked second, with more Category A and fewer Category C genes than either R9 protocol. For virulence-related genes (Fig. 1b), ONT20h R10 and ONT20h R9 detected similar totals (366 and 365, respectively), but ONT20h R10 had more Category A (247 vs. 235) and fewer Category C (23 vs. 34) genes. ONT48h R10 showed the lowest detection (352), suggesting longer runs may increase error rates. In plasmid detection (Fig. 1c), ONT20h R10 again performed best, with 36 genes detected and 26 (72.2%) in Category A, followed by ONT48h R10 (35 genes, 25 in Category A). Category C remained low across all analyses, indicating robust ONT performance, consistent with previous reports [7, 14]. For MGEs (Fig. 1d), none of the protocols detected Category A elements. R10 protocols recovered more MGEs in Category B (90–<100% identity), with ONT20h R10 reaching 28 (65.1%). R9 protocols detected more MGEs overall but with a higher proportion in Category C. All detailed genetic elements detected by each service are presented in Supplementary Material 2.

Although plasmid detection is not part of routine diagnostic workflows, it provides valuable insights into the dissemination of genetic material. These extrachromosomal structures often carry antimicrobial resistance and virulence-associated genes transferable through conjugation, promoting the persistence and spread of these markers across bacterial populations [5, 15]. Characterizing plasmids and other MGEs remains technically challenging, especially for elements containing repetitive regions that hinder accurate assembly with short-read technologies such as Illumina [5, 15]. Long-read sequencing provides better continuity across these complex regions, enabling more complete reconstruction of plasmids and other MGEs involved in bacterial evolution and resistance dissemination [3, 5–7]. However, even with the improved accuracy of the R10.4.1 flow cell and 20-h sequencing runs, the complete resolution of highly repetitive MGEs remains challenging [5, 7]. This limitation is particularly evident for small plasmids, which are often underrepresented in long-read-only assemblies due to biases introduced during ONT library preparation or constraints of the assembly algorithms. Although Rapid kits, as used in this study, are preferred over Ligation kits for recovering small plasmids, assemblers such as Flye and Raven still struggle to fully reconstruct them, an issue that can be mitigated by using hybrid assemblies that incorporate Illumina data [5, 16].

The comparison between phenotypic and genotypic AST results is presented in Table 2. To ensure consistency, only the 20 isolates sequenced with both R9.4.1 [7] and R10.4.1 (present study) flow cells were included in the analysis, which accounts for minor differences from the dataset reported by Cipriani et al. [7].

**Table 2:** Accuracy metrics of antimicrobial susceptibility testing (AST) based on genotypic methods on MRSA (methicillin-resistant *Staphylococcus aureus*) for the four protocols studied

Parameters	ONT R9 20 h	ONT R9 48 h	ONT R10 20 h	ONT R10 48 h
Sensitivity (CI 95%)	100% (88.43–100)	100% (88.43–100)	100% (88.43–100)	100% (88.43–100)
Specificity (CI 95%)	99.09% (95.04–99.98)	97.27% (92.24–99.43)	100% (96.70–100)	100% (96.70–100)
PPV (CI 95%)	96.77% (81.00–99.53)	90.91% (76.61–96.83)	100% (88.43–100)	100% (88.43–100)
NPV (CI 95%)	100% (96.67–100)	100% (96.61–100)	100% (96.70–100)	100% (96.70–100)
Accuracy	99.29% (96.08–99.98)	97.86% (93.87–99.56)	100% (97.40–100)	100% (97.40–100)
Cohen’s Kappa (CI 95%)	0.979 (0.938–1.00)	0.939 (0.870–1.00)	1.00 (1.00–1.00)	1.00 (1.00–1.00)

ONT: Oxford Nanopore Technologies; PPV: positive predictive value; NPV: negative predictive value; CI: confidence intervals

All protocols showed high diagnostic performance, with sensitivity and negative predictive value (NPV) reaching 100% across all conditions. For R10 flow cells, both 20 h and 48 h protocols achieved perfect agreement with phenotypic AST (accuracy 100%, κ = 1.0). For R9 flow cells, the 20 h protocol also performed well (accuracy 99.29%, κ = 0.979), whereas extending the run to 48 h slightly reduced specificity, positive predictive value (PPV), and overall accuracy (97.86%, κ = 0.939).

The accuracy of ONT sequencing has markedly improved with recent chemistry updates. Error rates, which averaged around 5–6% for V10 kits run on R9.4.1 flow cells, can now drop below 2% with V14 kits on R10.4.1, depending on basecalling models and overall data quality [5]. In addition to the structural improvements of the R10.4.1 flow cell, the motor enzyme that controls DNA translocation through the pore has been optimized to move the strand more slowly and uniformly, generating cleaner and more stable ionic current signals [5, 16]. In our analysis, basecalling continued to be performed with Guppy, which was updated from version 3.0.6 (used with R9.4.1) to version 7.0.9 with R10.4.1. The newer version implements advanced neural network models optimized for the R10.4.1 pore and V14 chemistry, providing more accurate interpretation of ionic current signals, particularly in homopolymeric and GC-rich regions [14, 16]. Thus, the observed performance gains result from the integrated updates to chemistry, flow cell design, and basecalling software. Future analyses could benefit from replacing Guppy with Dorado, the new ONT basecaller developed under a unified PyTorch framework. Dorado offers improved accuracy and throughput and optimized models for R10.4.1 flow cells [4].

The complete ONT20h workflow using the R10.4.1 flow cell demonstrated the best overall performance in this study, representing a promising option for complementary clinical diagnostics based on rapid whole-genome analysis. Its ability to produce accurate genomes in a single day offers a valuable time advantage over extended sequencing protocols, including hybrid approaches that combine short- and long-read data. This time efficiency is particularly relevant in public health contexts, such as hospital surveillance and outbreak investigations, where timely genomic information can guide infection control measures and therapeutic decisions [17, 18]. For instance, confirming or ruling out a suspected outbreak within 24–48 h of sample collection allows for immediate targeted interventions [18]. Furthermore, the rapid and precise detection of resistance determinants (e.g., *mecA*) and virulence factors can support antimicrobial stewardship by validating empirical therapy or prompting an early switch to a more targeted regimen. Moreover, our analyses were performed using clinical MRSA isolates rather than reference strains, reinforcing the practical applicability of this protocol under real-world diagnostic conditions. Although hybrid assemblies can yield marginal improvements in assembly accuracy [5, 16], they are generally more expensive and time-consuming, requiring the preparation of multiple libraries. Although not assessed in the present study, it would be interesting to evaluate whether the high-quality results achieved with the ONT20h R10 protocol are maintained without polishing steps.

Despite its strong performance, some limitations of the ONT20h R10 protocol should be acknowledged. First, our evaluation was restricted to a collection of MRSA isolates from a low-prevalence setting, which harbored a characteristic, limited repertoire of resistance genes (predominantly *mecA* and *blaZ*). While the protocol achieved perfect concordance with phenotypic AST for these determinants, its performance against a broader and more diverse array of resistance mechanisms warrants further investigation. Second, the applicability of this workflow to other bacterial species remains to be determined. Cipriani et al. (2025) [7] reported that the ONT20h R9 protocol showed reduced accuracy for *Klebsiella pneumoniae* ESBL-producing strains, suggesting that performance may vary according to genome complexity. Although the R10.4.1 chemistry represents a significant improvement, some studies indicate that short-read data (e.g., Illumina) may still be preferable for high-resolution analyses of small-scale variants (SNVs and INDELs, i.e., single nucleotide substitutions and small insertions or deletions) which can influence phylogenetic accuracy in some bacterial species [2, 4, 5]. However, a previous study from our group has demonstrated that ONT 20-hour protocols, even with the earlier R9.4.1 flow cell, can reliably support SNP-based outbreak analysis in *S. aureus*, reinforcing that the impact of sequencing technology on genomic resolution is organism-dependent [18].

## Conclusion

Overall, this work reinforces the potential of rapid long-read sequencing as a complementary approach for clinical microbiology and genomic surveillance. Broader clinical adoption of ONT-based protocols will depend on future efforts to improve cost-effectiveness, workflow standardization, and the accuracy of analytical tools and reference databases. Continued benchmarking across different bacterial species and sequencing chemistries will be essential to consolidate the robustness and scalability of these genomic strategies in real-world diagnostic settings [2].

## Supplementary Information

Below is the link to the electronic supplementary material.

Supplementary Material 1

Supplementary Material 2

## Data Availability

The authors confirm all supporting data, code and protocols have been provided within the manuscript. All genomes are publicly accessible in the GenBank database under the BioProject PRJNA658251 and PRJNA1368287.

## Abbreviations

AST: Antimicrobial susceptibility testing
MGE: Mobile genetic elements
MRSA: Methicillin-resistant Staphylococcus aureus
ONT: Oxford Nanopore Technologies
WGS: Whole genome sequencing
